# Continuous mutual improvement of macromolecular structure models in the PDB and of X-ray crystallographic software: the dual role of deposited experimental data

**DOI:** 10.1107/S1399004714017040

**Published:** 2014-09-30

**Authors:** Thomas C. Terwilliger, Gerard Bricogne

**Affiliations:** a Bioscience Division, Los Alamos National Laboratory, Mail Stop M888, Los Alamos, NM 87507, USA; b Global Phasing Ltd, Sheraton House, Castle Park, Cambridge CB3 0AX, England

**Keywords:** structure determination, model quality, data analysis, software development

## Abstract

Accurate crystal structures of macromolecules are of high importance in the biological and biomedical fields. Models of crystal structures in the Protein Data Bank (PDB) are in general of very high quality as deposited. However, methods for obtaining the best model of a macromolecular structure from a given set of experimental X-ray data continue to progress at a rapid pace, making it possible to improve most PDB entries after their deposition by re-analyzing the original deposited data with more recent software. This possibility represents a very significant departure from the situation that prevailed when the PDB was created, when it was envisioned as a cumulative repository of static contents. A radical paradigm shift for the PDB is therefore proposed, away from the static archive model towards a much more dynamic body of continuously improving results in symbiosis with continuously improving methods and software. These simultaneous improvements in methods and final results are made possible by the current deposition of processed crystallographic data (structure-factor amplitudes) and will be supported further by the deposition of raw data (diffraction images). It is argued that it is both desirable and feasible to carry out small-scale and large-scale efforts to make this paradigm shift a reality. Small-scale efforts would focus on optimizing structures that are of interest to specific investigators. Large-scale efforts would undertake a systematic re-optimization of all of the structures in the PDB, or alternatively the redetermination of groups of structures that are either related to or focused on specific questions. All of the resulting structures should be made generally available, along with the precursor entries, with various views of the structures being made available depending on the types of questions that users are interested in answering.

## Abstract 2

Macromolecular structures deposited in the PDB can and should be continually reinterpreted and improved on the basis of their accompanying experimental X-ray data, exploiting the steady progress in methods and software that the deposition of such data into the PDB on a massive scale has made possible.

## 1. Introduction   

### 1.1. Models and interpretation of macromolecular crystal structures   

The three-dimensional structures of biological macromolecules such as proteins, DNA and RNA are of high importance in many areas of biology and biotechnology. The structures of individual proteins and of various complexes (between proteins, between proteins and small molecules, and between proteins and nucleic acids) are all crucial for understanding how these molecules function to catalyze chemical reactions and to control metabolism, growth and development. Structures of proteins bound to candidate drug molecules have now assumed a central role in the discovery of lead molecules and their optimization as part of the development of new pharmaceuticals. The structures of natural and engineered proteins are crucial for rational engineering of these molecules to give them new functions or altered properties.

In most crystal structure determinations of macromolecules, the key product is a three-dimensional model. Most biological or biophysical interpretation of a molecule is performed using such a model as a representation of what is in the crystal (as opposed to using the original diffraction data or an electron-density map). This means that the details of the models affect how the structure will be interpreted and that knowledge of the uncertainties and limitations inherent in each model is crucial.

### 1.2. The Protein Data Bank is the definitive repository of macromolecular structures   

For the past 40 years, most of the models of macromolecules determined by crystallography have been deposited in the Protein Data Bank (PDB; wwPDB; Berman *et al.*, 2000 ▶; Bernstein *et al.*, 1977 ▶), an enormously important resource that includes macromolecular structures determined by nuclear magnetic resonance and electron-microscopy techniques as well. The PDB contains models representing nearly 90 000 crystal structures, with several thousand added yearly. Since 1 February 2008, it has been mandatory for the intensities (or amplitudes) of the diffraction data to be included in all new PDB depositions of crystal structures. This makes it possible, at least in principle, both to evaluate the models and to improve them.

The PDB is more than *a* repository of structural information for macromolecules. It is broadly viewed as *the* definitive or ultimate repository of this information. This distinction has several consequences. One is that worldwide users of the PDB, many of whom do not have in-depth knowledge about structure determination and its limitations, may use the models from the PDB as if they were unique, unambiguous representations of the structures of the corresponding macromolecules. Another is that any secondary repositories of structural models are not likely to reach a broad audience of users unless they add a great deal of value beyond that available in the structures from the PDB. A third is that the deposited structure in some measure represents a publication in its own right. While not currently recognized as a bibliographic citation, its identity (*via* a unique deposition code and also a registered digital object identifier or DOI), the validation procedures through which it has progressed (and has often been improved) and its provenance through recorded, named depositors give it a substantial weight in the academic community.

### 1.3. The current paradigm: one-time interpretation of the data for macromolecular structures   

For the first 20 years of the PDB (∼1970–1990), most structural biologists deposited only the three-dimensional models of the structures they had determined and not the crystallographic data, even though this was possible as early as 1976 (*e.g.* PDB entry 155c). There were many reasons why only the models were deposited. One was that the models were mainly used to interpret the functions and properties of the macromolecules (*e.g.* enzymatic mechanisms), and the crystallographic data used in the process were viewed as just a means to that end. Another was that crystallographers were considered the owners of the structures for some time after the structures were determined, with exclusive rights to their interpretation. For both of these reasons, once the model was obtained it was thought that the crystallographic data were almost superfluous. More recently, it became widely accepted that making some form of crystallographic data available is essential for the validation of structural information in each PDB entry (Baker *et al.*, 1996 ▶), and currently nearly all depositions of crystal structures into the PDB are accompanied by crystallographic data. Nevertheless, the vast majority of the worldwide use of data from the PDB remains focused on the models rather than on the crystallographic data. Correspondingly, access to information in the PDB is organized at the level of a PDB entry, which for crystallo­graphic data normally consists of a single model and any supporting data and metadata.

Currently, the typical procedure followed in the determination of a crystal structure is for a single person or group to collect X-ray diffraction data, obtain information on phases, create electron-density maps, interpret these maps in terms of an atomic model and refine the model to optimize its agreement with the diffraction data while maintaining conformity with the relevant *a priori* knowledge of macromolecular geometry. Once this procedure has been carried out, the resulting model and X-ray diffraction intensities are deposited as an ‘entry’ in the PDB and become available to anyone who wishes to use them. As mentioned above, it is almost always the models that are used at this stage. It is unusual for the diffraction intensities to be considered by the end users of information from the PDB.

In most cases, the interpretation of the crystallographic data made by the group that carried out the structure determination is the only one that exists today in the PDB. There is a mechanism allowing any given structure to be updated by its depositor(s), removing the existing entry and replacing it with a new one, but this is performed relatively infrequently. There is also a mechanism whereby anyone at all can use the deposited data, create a new model and deposit it as a new PDB ‘entry’; however, this is rarely performed because corrected entries of this kind must be accompanied by a peer-reviewed publication, and there is generally little incentive to publish such corrections for their own sake. The PDB itself recently carried out a ‘remediation’ of many of the entries in the data bank (Henrick *et al.*, 2008 ▶). This remediation was primarily aimed at achieving consistency in nomenclature and formatting of the data, although some errors were corrected as well.

### 1.4. Errors and uncertainties in three-dimensional models of macromolecules   

In general, the structures of macromolecules in the PDB are of very high quality and, taking into account effects of thermal motion and of disorder that are intrinsic to portions of a structure such as in loops, most features of these structures are well determined (Kleywegt, 2000 ▶; Brown & Ramaswamy, 2007 ▶; Dauter *et al.*, 2014 ▶). Nevertheless, there is always some (small) level of uncertainty in the coordinates of atoms in models representing macromolecular structures. Additionally, there may be (usually small) correctible errors in interpretation. Finally, the conceptual framework used to represent a macromolecular structure is itself limited, preventing a complete description of what is in the crystal.

At present, it is typical for diffraction data from crystals of macromolecules to be measured using position-sensitive digital imaging systems. Owing to limitations in measurement and the substantial damage that can be inflicted on crystals through irradiation by the X-ray beam, there are significant uncertainties in the relationship of the measured intensities of diffraction spots to an ideal undamaged structure (Borek *et al.*, 2010 ▶; Pozharski, 2012 ▶). Further, during each of the steps followed in determining the structures of macromolecules, decisions are made about how to treat the data, what outside information to include and what features to incorporate into the modeling process. These factors may complicate the interpretation of the electron-density maps and introduce uncertainty regarding some details of the final models, in particular in loops or other flexible parts of biological macromolecules. As a consequence of these and other factors, the three-dimensional models obtained from this technique typically do not fully explain the X-ray diffraction data (Lattman, 1996 ▶). This means that the features that are incorporated into the models do not represent everything that is present in the crystals.

Owing to the complexity of the analysis, small errors and omissions as well as ambiguities between alternatives are common in the interpretation of crystallographic data. Most crystallographic models contain at least some features that, given a thorough inspection, would generally be thought of as incorrect interpretations (Kleywegt, 2000 ▶). For example, these could include side chains in proteins that are placed in physically implausible conformations when the electron-density map clearly shows another conformation. The identification of small-molecule ligands bound to macromolecules and of their precise conformations and locations can be challenging and can lead to errors in interpretation (Weichenberger *et al.*, 2013 ▶). Additionally, crystallographic models typically do not fully describe the range of structures that are actually contained in a crystal (Furnham *et al.*, 2006 ▶). For example, parts of a molecule might be represented in one conformation when the data are more compatible with several conformations, and it might not be clear from the data exactly what those conformations are. From a more general standpoint, a specific pitfall in X-ray crystallography, compared with other techniques for investigating molecular structure, is the so-called ‘model bias’ problem, which is closely related to the phase problem, whereby electron-density maps calculated on the basis of an assumed model can show features present in that model that are in fact incorrect but arise from using that model as the sole source of phase information for the experimental amplitude data. Several technical advances have reduced the prevalence of model bias (see, for example, Read, 1986 ▶; Hodel *et al.*, 1992 ▶; Terwilliger, 2004 ▶; Pražnikar *et al.*, 2009 ▶), but its ever-present risk needs to be borne in mind at all times. Normally these errors and limitations decrease markedly if the X-ray data extend to high resolution, while they can be very severe for crystal structures determined with X-ray data extending to only low resolution (*e.g.* 3.5 Å or poorer; see, for example, Hunt & Deisenhofer, 2003 ▶). The errors and limitations in the representation of models of macromolecules can limit their utility in interpreting the biological roles of the molecules, for example how drugs bind to them or what effects changes in the chemical amino-acid sequence of a protein or base sequence in RNA have on their structures and functions.

### 1.5. Validation of structures: a constantly evolving task   

The limitations of crystal structures of macromolecules have been recognized for a long time, and there has been great effort in the macromolecular crystallography community to develop criteria for evaluating the resulting models. Very recently, a task force of structural biologists, in conjunction with the PDB, developed a comprehensive set of criteria for the evaluation of crystallographic structures (Read *et al.*, 2011 ▶). These criteria are now available for the structures in the PDB (Gore *et al.*, 2012 ▶), making it feasible to identify which amongst a set of related structures are of high quality. It might even become feasible to identify which of a set of structures is best suited for answering a particular biological question. From a broader perspective, however, it should be remembered that initial validation can only be carried out using the best-performing tools and methods available at the time when a new deposition is initiated. As those tools themselves improve, using them to re-examine older entries will typically reveal errors and resolve ambiguities that could not have been diagnosed at the time of the original structure determination and deposition. Validation is thus itself an intrinsically dynamic, evolving process, and does not guarantee that a PDB entry can remain static once validated upon deposition.

### 1.6. Why crystallographic data are rarely reinterpreted and redeposited in the PDB today   

Given the degree of uncertainty and the levels of error in crystallographic models that we have alluded to in §[Sec sec1.4]1.4, and the fact that new and improved methods for crystal structure analysis are constantly being developed, as will be discussed in §[Sec sec2]2, it might seem surprising that the macromolecular models in the PDB are not systematically updated so as to make each structure available in its most recent, highest-quality form as a matter of course. Several practical and sociological reasons are typically invoked to rationalize why this is infrequently performed.

One practical reason is that users of the PDB often do not have detailed knowledge about how to choose which model is the most appropriate one for their purpose. This means that if many models were available, there might be confusion about which one to use. Another practical reason is that if a series of models representing a structure were all to be deposited in the PDB and a set of papers were published describing features of the structure, then there could easily be confusion about which model in the PDB the description of the structure in a publication is associated with. All of the coordinates described in the publication would change slightly even upon simple re-refinement of a structure in any case. A reader of a publication would then have to refer to the exact structure that the authors used at the time if they wished to compare it with the published information. A third practical problem is that updated versions of structures could use different nomenclatures or have different numbers of atoms in the model, for example if some of the structures were incomplete. These simple changes would make comparisons between publications and any updated structures more difficult. A fourth practical reason is that it requires a great deal of work to deposit a structure in the PDB. The structure and all of the data and metadata that go with it must be deposited, validated and checked for accuracy. To perform this for a large number of structures would be a huge undertaking.

We note that these practical arguments focus on activation barriers that are no longer major challenges. Problems with multiple models and confusion in cross-referencing models and publications could be readily solved by standard procedures of version control and by providing fine-grained descriptors for the inter-relationships among PDB entries that could be used in publications. These problems and their solutions are generic in numerous scientific fields and there has been considerable investment over the past decade, under the headings of ‘Cyberinfrastructure’ (US) or ‘e-Science’ (Europe), in the development of tools to implement solutions capable of addressing these issues.

A key sociological reason why models in the PDB often remain static is that structural biologists typically regard a structure as their personal scientific contribution. This view of a structure has consequences both for the scientist or group that determines a structure and for all others. The scientist who determines a structure has invested in its correctness and completeness because they have performed all of the work necessary to determine the structure and have deposited and published it. They may also have published other papers based on this interpretation of the structure. There is therefore substantial motivation not to update the structure unless it is seriously deficient, often because of staff turnover and lack of resources to revisit the work performed by a scientist who has moved on to a new position. This view of a structure also has implications for other scientists. If another scientist updates a structure on the basis of the original data and deposits an improved structure, using the ‘REMARK 0’ mechanism to clearly link it to the initial PDB entry that it is intended to override, this could easily be taken as a criticism of the work of the original depositor, even if the intent were solely to build on and possibly add to the work of the original depositor.

Finally, a composite of practical and sociological factors in making the re-deposition of improved models into the PDB rarer than expected could be described as ‘PDB error fatigue’. Crystallographers know that it is easy to find errors in PDB entries but arduous to remediate them, and therefore become too easily resigned to the persistence of the static archive paradigm.

## 2. Experimental data deposition into the PDB as the common foundation for both the rapid progress in computational methods and the improvement potential of PDB models   

In the foregoing discussion we have mentioned the continuous development of new and improved crystallographic methods as if it were an autonomous background process separate from the growth of the PDB and independent of the precise nature of its contents. It is, however, of the utmost importance to recognize that this is not the case and that the discussion of future data-deposition policies for the PDB should take into account the tight mutual interdependence of methods developments and the PDB contents.

The main theme of this article is indeed the ‘virtuous circle’ involving software improvements being enabled by enriched PDB deposition contents, which in turn make it possible to improve the deposited structure models by re-analyzing the same original experimental data deposited along with the initial models. In this section we want to underline the profound impact that the co-deposition of ‘structure-factor’ data, along with the models of macromolecular crystal structures derived from them, has had on the whole field to support the idea that any decision regarding the archiving of further experimental data by the PDB should be considered in that full light.

One can hardly overstate the role of the large-scale availability of experimental X-ray data through their deposition into the PDB in fuelling the remarkable progress of computational crystallographic methods and software. Several decades ago, when macromolecular structure refinement was still being carried out by least-squares methods without the warning bell of cross-validation through the free *R* factor (Brünger, 1992 ▶), the resulting models could potentially be affected by unquantifiable degrees of overfitting that could never be assessed if experimental data were not deposited. A slow process of methods improvement in the refinement area started with the move from least-squares to maximum-likelihood targets (Bricogne & Irwin, 1996 ▶; Murshudov *et al.*, 1996 ▶; Pannu & Read, 1996 ▶). This was later supplemented by the use of better restraints and better enforcement of NCS (Smart *et al.*, 2008 ▶, 2011 ▶, 2012 ▶; Nicholls *et al.*, 2012 ▶; Headd *et al.*, 2012 ▶). Developments in other areas of computational crystallo­graphy yielded better methods for the detection of twinning and the reassignment of space-group symmetry (Lebedev *et al.*, 2006 ▶, 2012 ▶; Zwart *et al.*, 2005 ▶, 2008 ▶; Le Trong & Stenkamp, 2007 ▶, 2008 ▶; Stenkamp, 2008 ▶; Poon *et al.*, 2010 ▶; Zhang *et al.*, 2012 ▶) as well as better schemes for bulk-solvent correction (Fokine & Urzhumtsev, 2002 ▶; Afonine *et al.*, 2013 ▶), density modification (Terwilliger, 2000 ▶; Cowtan, 2010 ▶; Skubák & Pannu, 2011 ▶) and phase combination (Skubák *et al.*, 2010 ▶). All of these improvements have contributed to the production of less and less biased maps and of models with better and better molecular geometry. The resulting improved maps, for example, have become increasingly capable of showing whether the ligands present in a model are supported by the crystallographic data (Pozharski *et al.*, 2013 ▶). Finally, new tools for automated model building (Terwilliger *et al.*, 2008 ▶; Langer *et al.*, 2008 ▶; Cowtan, 2006 ▶, 2012*a* ▶,*b* ▶) and for interactive examination of models and electron-density maps incorporating powerful refinement and rebuilding capabilities (Jones *et al.*, 1991 ▶; Emsley *et al.*, 2010 ▶) have changed the task of detecting and correcting model errors by hand and eye into a highly automated procedure.

The unique resource provided by the experimental data associated with PDB entries has played a crucial role in the invention and validation of these new methods. Before the deposition of such data, the scope of the investigation of new ideas in X-ray methods was often limited to a few in-house data sets. As the validation of any new method demands that its performance be assessed on a ‘test set’ of data not used as part of any ‘learning set’ that guided the development of the method itself, such validation was necessarily limited and took place slowly in the user community after the new software had been released. The deposition of experimental data into the PDB created a radical change of development conditions for new methods and software, providing large-scale collections of data sets making it possible to thoroughly test and validate any new approach before its release to users in the form of new software.

This consolidation of the iterative process of methods improvement has played a major but perhaps understated role in the huge effort towards automation that was spurred by the advent of structural genomics at the turn of the century, leading to today’s integrated systems for (quasi-)automated structure determination such as, for example, the *CCP*4 and *PHENIX* software packages (Winn *et al.*, 2011 ▶; Adams *et al.*, 2010 ▶). It is now possible in many cases to carry out all of the steps from integration of diffraction intensities to interpretation of the data in terms of a nearly final atomic model in an automated fashion (see, for example, Afonine *et al.*, 2012 ▶), with only the very last steps of checking the structure, fixing small errors and interpreting regions in the electron-density map that involve multiple conformations still being performed manually.

The idea that the interpretations of structures represented in the PDB should be updated as improved methods or information became available has been discussed for some time. Kleywegt *et al.* (2004 ▶) noted that

…in the long term the community will probably have to face the issue of whether the structural database should be static or dynamic. As methodology improves, it seems likely that re-refinement of older models (either on a case-by-case basis, or as one large-scale project) might provide better models and, hopefully, increase our understanding of the chemistry and biology of the molecules under study.

Later, Joosten, Womack *et al.* (2009 ▶) expanded on this, noting that

…improvements in crystallographic software and validation tools, combined with the deposition of X-ray data into the PDB, have enabled the development of automated re-refinement protocols…which can improve most structure models compared with their initially deposited form….

On the basis of this observation, they envisioned that

…users of the PDB and software developers will greatly benefit…as it will turn what was previously a static archive of frozen models into a repository of self-improving results through the steady progress in methods developments it will catalyze…. Depositors will also benefit…because it will make their structural results more future-proof, leading to more citations and to higher visibility.

These ideas create a vision of how powerful the continuous updating of structures could become if the paradigm of ‘one pass through the data plus archiving’ were abandoned or slowly superseded, and of how improvement in methodology constitutes a process closely interrelated to the improvement of results themselves, both being fuelled by the accumulation in the PDB of original experimental data accompanying the results derived from them.

The task of automating not only structure refinement but also the detection and correction of various modelling errors or shortcomings has been the focus of particularly intense effort, culminating in the *PDB_REDO* project (Joosten, Salzemann *et al.*, 2009 ▶; Joosten *et al.*, 2011 ▶, 2012 ▶) demonstrating the operational feasibility of structure improvements on a whole-PDB scale and showing that the various sources of reticence towards updating PDB entries, as reviewed in §[Sec sec1.6]1.6, are now due for fresh rethinking. The automated *PDB_REDO* system carries out validation, model improvement and error checking on PDB entries that contain X-ray data and provides updated models that often constitute improvements over the original PDB entries as judged by agreement with crystallo­graphic data and with expected geometry. Although the *PDB_REDO* system is not yet capable of fixing all readily apparent errors (Dauter *et al.*, 2014 ▶), procedures for automated crystal structure interpretation continue to improve and it seems likely that in the near future fully automated procedures for structure determination of macromolecules may be applied in many cases.

Before making the detailed case in favor of applying the *PDB_REDO* approach to the continuous improvement of the contents of the PDB itself on the basis of its ‘structure-factor’ data archive, it is worth pointing out that the article by Joosten, Womack *et al.* (2009 ▶) cited earlier envisioned an extension of the scope of the continuous improvement process to the archiving of raw experimental diffraction images, expecting that this would provide the same stimulus towards improvements in the corresponding software as the deposition of ‘structure-factor’ data provided towards the improvement of refinement protocols and their automation. These authors argue that

In the same way that deposited coordinates are only the best results that could be obtained from the deposited X-ray data by the refinement protocols available at the time and are therefore improvable as these protocols become more sophisticated, those deposited X-ray data are only the best summary of sets of diffraction images according to the data-reduction programs and practices available at the time they were processed. Just like refinement software, those programs and practices are subject to continuing developments and improvements, especially in view of the current interest and efforts towards better understanding radiation damage during data collection and in taking it into account in the subsequent processing steps.

We will return to this topic in §[Sec sec4.2.2]4.2.2.

## 3. Continuous improvement of macromolecular crystal structures   

We suggest that despite the technical challenges that the task is bound to present, the structural biology community now can and should undertake systematically to improve the tens of thousands of models in the PDB that represent macromolecular crystal structures and make them available within the PDB itself. A change of focus from a fixed interpretation of a crystal structure to an ever-improving modeling of that structure is technically feasible and is highly desirable, as this will improve the quality, utility and consistency of the structures in the PDB. We propose that the PDB should maintain both original interpretations and reinterpretations of macromolecular structural data as integral and widely accessible elements of its repository.

While most of the discussion below focuses on automation, as this will be required by the sheer magnitude of the task of continuously updating the contents of the PDB, it is worth pointing out that model errors or inadequacies that are not correctable in an automatic manner are regularly found by investigators but rarely corrected, for the reasons described in §[Sec sec1.6]1.6. Lowering the activation barrier to such ‘crowdsourcing’ contributions to improvements that currently elude automation is another area where the deployment of modern information technology could have a beneficial impact on improving the PDB contents. Use of these approaches could motivate crystallographers to not only write papers about shortcomings in models, but also deposit their improvements. The paper by Smart *et al.* (2012 ▶) is a good example of a description of a methodological advance that made it possible to produce better models for two PDB entries from the associated experimental data and where these improved models were deposited as new entries linked to the original entries *via* the REMARK 0 field.

### 3.1. Reinterpretation of the data is feasible   

Automation of structure-determination algorithms and the availability of crystallographic data for most of the macromolecular structures in the PDB have made it feasible to systematically reinterpret these structures. The full-scale validation of crystal structures in the PDB (for example using the Uppsala electron-density server; Kleywegt *et al.*, 2004 ▶) shows that automated procedures can reproduce many of the validation analyses needed to reinterpret structures, including the comparison of models with crystallographic data. The re-refinement and model correction performed by *PDB_REDO* further shows that improvement of models can be systematically carried out. These developments, along with the continuous and dramatic improvements in the automation of macromolecular structure determination, make it feasible to systematically reinterpret macromolecular crystal structures.

### 3.2. Reinterpretation of the data is desirable   

There are many reasons why it is highly desirable to reinterpret crystallographic data. At a basic level, reinterpretation with modern approaches can easily correct small but clear errors in existing structures. Certainly, if two interpretations of a structure are identical except that one of them has amended some clearly incorrect features in the other, then it would be advantageous to use the corrected structure in any further analyses involving that structure.

Also at a basic level, if a consistent set of procedures were to be applied to the structure determination of all structures in the PDB, then the resulting models would have a higher degree of consistency than is currently the case. This would reduce the number of differences between models in the PDB that are due only to the procedures and not to genuine differences in the crystal structures. A good analysis of how the exact methods used can affect a crystal structure (in this case the bond lengths involving the copper in this structure) was described some 20 years ago (Guss *et al.*, 1992 ▶).

At a second level, a reinterpretation of a structure with new algorithms or new outside information might yield structural information that was not present in an initial structure. This could include structures for less well ordered regions (‘floppy bits’) or for small-molecule ligands that could not be modeled in the initial structure.

At a more sophisticated level, the entire formalism of how crystal structures are represented is likely to change over time. At present a structure is typically described by a single set of coordinates, occasionally containing a few regions that are represented by multiple conformations. It is arguable that in the future most macromolecular crystal structures will be presented in the form of ensembles of models. These could aim at giving a coverage of the diversity of structures present among all of the copies of a molecule in a crystal, or at representing the uncertainties attached to a macromolecular structure by exhibiting how many variants of it would remain compatible with the observed data.

At a very sophisticated level, the most useful model for a particular analysis may depend on what the analysis is intended to achieve. Let us assume, for example, that the goal is to determine the structural differences between a pair of proteins that are crystallized in the same crystal form in the presence and absence of a small-molecule ligand. If these two structures are determined and refined against the crystallo­graphic data independently, there are likely to be many small differences between the resulting structures simply owing to minor differences in procedure. In contrast, if the two structures are refined together and only model differences that are strictly required by differences in the crystallographic data are allowed, then the structures will be much more similar and the differences will be much more likely to be meaningful (see, for example, Vojtechovský *et al.*, 1999 ▶). Although such a pair of jointly determined structures may have the most accurate differences in structure, they may or may not have the most accurate individual structures. This example suggests that it may be desirable to have custom sets of structures where all of the structures in a group are modeled together so as to have the most accurate set of comparisons of these structures.

Also at a sophisticated level, the crystallographic models currently in the PDB may have been based on structural information from earlier structures but never from later ones. If the entire PDB is reinterpreted, this no longer has to be the case. An approach related to joint refinement of structures is the increasingly important method of using a high-resolution structure as a reference model in the refinement of a low-resolution model (Smart *et al.*, 2008 ▶, 2012 ▶). This approach essentially uses the expectation that the low-resolution structure is generally similar to the high-resolution structure one and that it only differs in places where the low-resolution crystallographic data require it to be different. Such an approach can now be applied retrospectively to structures in the PDB.

### 3.3. Reinterpretation is desirable even though the PDB is growing rapidly   

It might be argued that because the PDB is growing so rapidly there is little point in worrying about the structures that have already been deposited. It is indeed very likely that today’s nearly 90 000 structures will soon constitute only a small fraction of the total contents of the PDB. On the other hand, the structures that have already been determined represent a tremendously important set of structures, as most of these structures were chosen based on their biological importance. Despite advancements in structure-determination methodology, carrying out the gene cloning, expression and purification of proteins, crystallization and X-ray data collection on these tens of thousands of structures all over again will remain prohibitively expensive for a very long time: re-determining them all today from the beginning might cost in the range of $1–10 billion, even using current high-throughput approaches such as those used in the field of structural genomics, which have a cost per structure of about $70 000 (Terwilliger *et al.*, 2009 ▶). Consequently, it is extremely important, indeed imperative, to have the best representation of today’s structures, not just of those that will be determined in the future.

### 3.4. Validation and evaluation of reinterpretations of crystal structure data   

One of the key reasons why it is appropriate to undertake the continuous reinterpretation of macromolecular crystal structure data now is that comprehensive validation tools suitable for widespread deployment have become available (Pozharski *et al.*, 2013 ▶). The validation suite developed for the PDB provides a way to evaluate a structure for geometrical plausibility and fit to the data and to compare these metrics with values for other structures in the PDB determined at similar resolution. This means that systematic criteria are available for the evaluation of new models relative to existing ones.

It is important to note that the validation criteria currently used are not direct measures of the accuracy of the structure if accuracy is defined in terms of the positional uncertainty in the coordinates of the atoms in the model: rather, the validation criteria are indirect indicators of that accuracy. For example, perhaps the best known validation criterion is the Ramachandran plot, which displays the distribution of ϕ–ψ angles along a polypeptide chain of a particular protein in such a way as to allow its easy comparison with those of thousands of well determined protein structures (Ramachandran *et al.*, 1963 ▶). A structure with an unlikely Ramachandran distribution is unlikely to be accurate, but there is no simple correspondence between these measures.

Although metrics for structure quality are available, there is not any single metric that can be used effectively to rank structures. For any given metric there is a range of values of that metric for structures in the PDB. For many geometrical criteria there is also an underlying range of values from small-molecule structures. A particular structure may be in the most common range for some criteria and an outlier for others. Having unusual values for some metric does not necessarily mean that the structure is incorrect. That could be the case, or it could be the case that the structure has an unusual feature. However, structures with many unusual values for many criteria are generally found to have serious errors (Kleywegt, 2000 ▶). Another type of metric is the Cruikshank–Blow Diffraction Precision Index, which gives an overall estimate of uncertainties in atomic coordinates (Cruickshank, 1999 ▶). While this is a useful measure of quality, it does not differentiate between different types of errors (inadequacies in the model representation itself compared with coordinate errors, for example). Overall, existing validation metrics can be used to identify whether a structure is generally similar in quality to other structures in the PDB. It is likely that structures with better metrics overall are generally more accurate than structures with worse metrics, although this has not been demonstrated except for extreme cases.

In addition to quality metrics, it may be important in some cases to evaluate model quality by considering the information that is used in crystal structure determination. As a simple example, some piece of experimental information (for example anomalous diffraction data) might be used in the refinement of one model but not in another. Although this might not change the overall metrics substantially, the structure obtained using the greater amount of experimental information might generally have smaller coordinate errors (provided that the additional experimental data are accurate and not from a crystal with serious radiation damage). Similarly, if two structures are determined using nominally the same data but one structure is refined using only a subset of the data and the other using all of the data, the one obtained with all of the data has the potential to be the more accurate of the two.

It is also important to further develop the metrics for structure quality, along with an understanding of the relationship between quality metrics and coordinate uncertainties. Also critically important will be the development of metrics that identify the uncertainties in features in electron-density maps. Such metrics would greatly strengthen the ability to distinguish features of models that must be present to be consistent with the data from those that simply can be present and are consistent with the data. For structures at low resolution, the latter situation can lead to models that contain features that are not actually present in the crystal. Another kind of metric that will be important to develop is an assessment of whether or not the atoms in a model correspond to the correct atoms in the structure. For example, metals, ligands and segments of the macromolecule in models may be incorrectly identified (Kleywegt, 2000 ▶; Zheng *et al.*, 2008 ▶; Weichenberger *et al.*, 2013 ▶). Current automated procedures can sometimes fix register alignments of a macromolecule but ordinarily cannot fix incorrect ligand assignments, for example (Joosten *et al.*, 2012 ▶).

### 3.5. Deciding which structure or group of structures should be used in an analysis   

As there is no single measure of the quality or accuracy of a structure, only metrics that collectively indicate something about that quality, it is not simple to decide which model is the best representation of a particular structure when several are available. Also, as mentioned above, the structure or set of structures that is most informative may even depend on the question that is being asked.

A useful approach to deciding what structure to choose may be to start with the scientific question that the structure is expected to help to address. Some questions could be enumerated in advance and grouped according to the kind of information that is needed to answer them. Others might require a custom analysis of what structures are available before the most appropriate one can be identified. Still others might require a custom redetermination of certain structures in order to best be adequately answered.

Questions that do not depend on the fine details of a model might include ‘What is the overall fold of this protein?’ and ‘Are these two molecules similar in conformation?’ Such questions can be answered for a protein molecule without requiring a detailed knowledge of the conformations of its side chains and even with the main-chain atomic coordinates being somewhat approximate, as differences of less than about 1.5–2 Å would not substantially change the answers. In other words, answers to such questions could be provided on the basis of any model that is not grossly inaccurate.

Another set of questions, perhaps the most common set, would be more dependent on the overall correctness of a model. For example, ‘What is the buried contact area between the proteins in this complex?’ would depend on the positioning of main and side chains in the contact region of the two proteins. If two models for this complex based on the same data were available, it is likely that the model that is more generally correct would be the more useful of the two. Similarly, if two models that are nearly identical are available, where one had clearly incorrect features while the other did not, the one without obvious errors would be most likely to be more useful. A related approach would be to start with the original model for a given structure. Then if another model for the structure was available that had some clearly better quality metrics and similar or better quality for all other metrics, and the new model was as complete as the original, that new model might be more likely to be useful.

Other questions might depend on the details of a model. For example, ‘What is the coordination of this Fe atom?’ depends on interatomic distances and correct placement of the Fe atom and the side chains coordinating the iron. The model that best answers this question probably will have had a careful consideration of the positions of the iron and coordinating side chains and their agreement with both the crystallographic data and plausible geometry. If the oxidation state of the iron is known, then the refinement would be expected to include appropriate geometry and distances for that state. Another question depending on the details of a model is ‘What is the distance between this arginine side chain and this glutamate side chain?’ Answering this question requires knowing whether these two side chains are largely in single conformations, and if so, what these conformations are. A structure where these two particular side chains agree closely with the electron-density map is more likely to be useful in answering this question than one where they do not.

Still other questions might depend on the relationship between one or more models and require a custom or grouped analysis. ‘What is the variability in side-chain conformations depending on temperature?’ requires a comparison of several structures. Most likely, a useful comparison would involve an analysis of several structures performed using the same refinement and modeling techniques for all of the structures.

Another, completely different, approach to choosing which model to analyze will be to use all of them. Nearly all structures will have some useful information. By analyzing all of the models and all of their agreements with geometrical considerations and with the crystallographic data, it might be possible to identify what is known and what is not known in this structure. A more general approach would be (as mentioned above) to deliberately create many models representing what is in the crystal and to use the variation among these models (or ensembles) as an estimate of the uncertainty in the models.

### 3.6. How a user can find the right model or models to analyze   

If there are many models for each crystal structure, then attention should be given to the need to provide users with an easy way to find the model or models that best suit their needs. Based on the discussion above, one way to do this would be to have different views of the PDB depending on the question that is being asked. For users who do not have any specific question in mind or do not want to share their question, there might be standard views. One of these might be similar to the current view of the PDB, showing all original structures along with structures revised by their authors. Another, as discussed above, might be a view of the original model or the model most clearly improved over the original. Other views might include groups of structures that were all redetermined together or groups of structures redetermined with particular questions in mind.

As different models for a crystal structure may be useful for different purposes, it is important that the original structural model as well as subsequent reinterpretations of the data be readily available for analysis. An important role can be played by the PDB in archiving a series of interpretations of each crystal structure and facilitating the retrieval of suitable models for the purposes of varied users. In particular, the metadata associated with each model and crystal data should include information allowing a careful decision to be made about which model to use.

## 4. Generating and storing interpretations of crystal structure data   

### 4.1. Generating new interpretations of crystal structure data   

The generation of new interpretations of crystal structure data could be carried out in a variety of ways. Individuals could continue to reinterpret their own data and could reinterpret the data of others, particularly structures in which they have specific interest and expertise. This ‘crowdsourcing’ mechanism alluded to earlier could make major contributions to remediation tasks that are beyond the reach of automated procedures. Additionally, however, large-scale efforts (such as *PDB_REDO*) could systematically reinterpret crystal structure data using standardized procedures. Some efforts might focus on individual structure redeterminations, while others might focus on joint refinement of groups of structures. As mentioned earlier in §[Sec sec3.2]3.2, an important feature of such large-scale efforts would be that the procedures would be essentially identical for all structures, lending an increased consistency to that set of structures as a whole. Both small-scale and large-scale efforts might create multiple reinterpretations of any given structure.

A key outcome of this process is that reinterpretation of a specific crystallographic data set would no longer be considered to be a statement that the original model is in error: rather, it would be seen as part of a process of continuous improvement of all models in general.

An important aspect of generating new interpretations of crystal structures is the checking and storage of the data, models and metadata associated with the new interpretations. As mentioned above, PDB depositions currently require a substantial investment of effort for an individual depositor. This will likely remain the case in the future. For large-scale efforts, however, the corresponding process might be highly automated, perhaps with only a component of manual checking to identify situations that were not handled properly by automated procedures. The availability of existing models that can be used as a comparison with any new models for a particular structure could facilitate the development of a highly effective process for identifying any errors or omissions in new models. This could in turn allow a fully automated process for continuous improvement of models for a structure.

In the long term, it is essential that reinterpretations of structural data be stored as an integral part of the PDB so that these interpretations are widely and permanently accessible. The storage of several or even many models for each structure represented in the PDB presents a significant challenge for the short term, and as a temporary measure other alternatives could be followed. At present, the models created by *PDB_REDO* are stored locally, for example. Such a system would be able to make models available only as long as the local servers were supported. This would mean that some data could be available for a limited period of time only. Though not optimal, this could still be useful, although on an interim basis only. A particular model might have a limited lifetime during which it is an important source of information (and after which some other, better, model serves the same purpose). The significant disadvantage of any system that is not centralized is that it may not be possible to reproduce a particular analysis of the entire PDB at a later date. The counter argument is that it is not always necessary to be able to reproduce an analysis exactly, only to reproduce the process that would generally give a similar overall result. However, such arguments risk excusing procrastination, and while the immediate usefulness of having locally stored versions of reinterpreted structures cannot be denied, it is essential in the longer term that the archiving of these reinterpretations should become part of the remit of the PDB.

### 4.2. Data and metadata needed to facilitate reinterpretation   

The PDB already accepts essentially most of the information that would be important in facilitating reinterpretation of macromolecular crystal structures. Information that the PDB accepts includes crystallographic data, model information and metadata on the procedures used. As discussed in the accompanying articles, there are many strong arguments for storing raw crystallographic images as well.

#### 4.2.1. Overall metadata   

There are several types of metadata that are very helpful in understanding what was performed in a structure determination and that can be crucial for carrying out a new structure determination based on the original data. These include the following.

(i) What information was used to obtain the final model (crystallographic data, other structures, restraints libraries)?
(ii) What type of model was used (*e.g.* TLS or atomic ANISOUs with restraints; solvent representation)?
(iii) What general approaches were used to determine the structure represented by the model (molecular replacement, SAD or MAD phasing)?
(iv) What are the values of all the validation metrics?

In addition to these metadata, the model and a raw or a processed form of the data themselves can be collected:

(v) What are all the values of all of the parameters in the model and the estimates of their uncertainties?
(vi) What are the values of all of the crystallographic data used to determine the model?

As mentioned above, the raw crystallographic data are currently not normally archived by the PDB. However, data that have been subjected to minimal processing (for example, where measurements that may or may not be duplicates of each other depending on the space group of the crystal are not averaged) can be deposited and are themselves substantially more useful than fully processed crystallographic data.

All of these metadata can be recorded along with any other specialized information about the structure, such as:

(vii) What are all the components in the crystallization droplet, including any chemical connectivities and modifications, and stoichiometries?
(viii) What existing structures were used as templates in structure determination and how were they modified?

#### 4.2.2. Crystallographic data and metadata that are not consistently deposited in the PDB at present   

To facilitate systematic reinterpretation of crystal structures, the structural biology community would need to consistently deposit all of the information listed above. At present, most of this information is required for PDB deposition. Items that are not required but that would make full reinterpretation feasible would include raw diffraction images and all diffraction data, including data collected at multiple X-ray wavelengths and data from heavy-atom derivatives.

Raw diffraction images, whether exactly as collected or processed to conform to standardized image formats, are an important source of information about a crystal structure because they contain information about disorder in the crystal that is discarded during integration and the calculation of diffraction intensities. They also may contain information about multiple crystals that may have been in the X-ray beam. Most importantly, they contain the diffraction data in a form unaffected by how it is subsequently processed to produce the current type of ‘structure-factor’ data, which involves a very large number of decisions about space group, crystal shape, absorption, decay and diffraction physics.

It is very likely that the methods of interpretation of raw diffraction images will improve in the future, allowing more accurate interpretations of crystal structures, so that the preservation of this information will make an important contribution to the future improvement of models of crystal structures. The same ‘virtuous circle’ of mutual improvements in software and final results can be expected to establish itself as described in §[Sec sec1]2, fuelled this time by the large-scale availability of full sets of raw diffraction images and offering the opportunity to revisit long-held views about such fundamental operations as spot integration and data scaling that may indeed be overdue for a reappraisal.

A second type of data that is not consistently preserved consists of the multiple crystallographic data sets that often need to be used in structure determination. In many cases, only the crystallographic data against which the deposited model was refined are preserved, and accessory data collected at multiple wavelengths or from heavy-atom derivatives to obtain phase information are not deposited. As these crystallographic data contain information about the same or very closely related structures, their preservation will very likely be helpful in obtaining improved models of these structures. They would also play the same dual role of enabling faster improvement of experimental phasing methods and software. A striking example of a significant loss of experimental phase information in conventional approaches to the processing of multiwavelength data that can be reclaimed by revisiting original images with more sophisticated software is the common neglect of the anisotropy of anomalous scattering (Schiltz & Bricogne, 2008 ▶).

## 5. Conclusions   

The transformation of the PDB from a static archive to a dynamic body of continuously improving results is an idea whose time has come. The regular updating of models of macromolecular structures is now becoming feasible and the availability of systematically re-analyzed models will improve the overall quality and consistency of models across the PDB, allowing better biological and engineering conclusions to be drawn from them.

We have deliberately emphasized that this continuous improvement process is mediated by the improvements in methods and software that are made possible by the archiving of increasing amounts of experimental data. The next step in this direction should be the archiving of raw diffraction images.

There remain some challenging aspects to this task, including the choice of views of the PDB contents adapted to the diverse categories of users of macromolecular structures, the development of procedures for storing and checking these contents and the provision of resources to make these models available. The computer science knowhow is however sufficiently established and available to meet these challenges. The prospects therefore appear highly favorable for some implementation of continuous improvement and updating to be carried out without delay.
